# Extracellular Volume Fraction Calculated Using Contrast-Enhanced Computed Tomography as a Biomarker of Oxaliplatin-Induced Sinusoidal Obstruction Syndrome: A Preliminary Histopathological Analysis

**DOI:** 10.1155/2023/1440257

**Published:** 2023-02-14

**Authors:** Kengo Kai, Takeomi Hamada, Yuichiro Sato, Masahide Hiyoshi, Naoya Imamura, Koichi Yano, Takuto Ikeda, Akiko Ichihara, Shogo Ogata, Narantsog Choijookhuu, Yoshitaka Hishikawa, Ayumu Hosokawa, Atsushi Nanashima

**Affiliations:** ^1^Department of Surgery, Faculty of Medicine, University of Miyazaki, Miyazaki, Japan; ^2^Department of Anatomy, Histochemistry and Cell Biology, Faculty of Medicine, University of Miyazaki, Miyazaki, Japan; ^3^Department of Diagnostic Pathology, Faculty of Medicine, University of Miyazaki, Miyazaki, Japan; ^4^Department of Clinical Oncology, University of Miyazaki Hospital, Miyazaki, Japan

## Abstract

**Background:**

Oxaliplatin (OX)-based chemotherapy induces sinusoidal obstruction syndrome (SOS) in the nontumorous liver parenchyma, which can increase the risk of liver resection due to colorectal liver metastasis (CRLM). The extracellular volume (ECV) calculated from contrast-enhanced computed tomography (CT) has been reported to reflect the morphological change of hepatic fibrosis. The present retrospective study aimed to evaluate the ECV fraction as a predictive factor for OX-induced SOS.

**Methods:**

Our study included 26 patients who underwent liver resection for CRLM after OX-based chemotherapy with a preoperative dynamic CT of appropriate quality. We investigated the relationship between the pathological SOS grade and the ECV fraction.

**Results:**

Overall, 26 specimens from the patients were graded with the SOS classification of Rubbia-Brandt et al. as follows: grade 0, *n* = 17 (65.4%); grade 1, *n* = 4 (15.4%); and grade 2, *n* = 5 (19.2%). No specimens showed grade 3 SOS. In a univariate analysis, the ECV fraction in grade 0 SOS was significantly lower than that in grade 1 + 2 SOS (26.3 ± 3.4% vs. 30.6 ± 7.0%; *P* = 0.025). The cutoff value and AUC value of the ECV fraction to distinguish between grades 0 and 1 + 2 were 27.5% and 0.771, respectively.

**Conclusions:**

Measurement of the ECV fraction was found to be a potential noninvasive diagnostic method for determining early-stage histopathological sinusoidal injury induced by OX-based chemotherapy.

## 1. Introduction

The liver is the most common site of metastasis of colorectal cancer, and liver metastasis is a major contributor to mortality in patients with this disease. Liver resection currently remains the treatment that offers the best chance of long-term survival to patients with colorectal liver metastasis (CRLM) and has become the standard of treatment in resectable cases [1, 2]. Systemic chemotherapy is the standard treatment for patients with unresectable CRLM. Perioperative chemotherapy has been proven to effectively downsize metastases, improving disease-free survival in patients with resectable disease [3]. On the other hand, systemic chemotherapy for CRLM has been demonstrated to cause injury to the nontumoral liver parenchyma [4, 5]. Oxaliplatin (OX) can induce hepatic sinusoidal obstruction syndrome (SOS), which can lead to thrombocytopenia and severe liver dysfunction [6] and a higher rate of postoperative morbidity [7]. Therefore, it is important to establish an accurate method with which the histopathological change of the liver caused by SOS can be evaluated. The development of a noninvasive imaging technique would enable wide screening and closer monitoring of patients who are at risk for SOS. So far, few effective imaging findings have been shown to reflect the parenchymal changes in OX-induced SOS. Overman et al. [6] reported that spleen size correlates with increasing grade of hepatic sinusoidal injury. O'Rourke et al. [8] also demonstrated that ferucarbotran-enhanced MRI could predict the severity of chemotherapy-associated hepatic cellular injury. As a novel candidate quantitative diagnostic method for SOS, we focused on the measurement of the extracellular volume (ECV) fraction on contrast-enhanced computed tomography (CE-CT), which several studies have reported to have potential application in the diagnosis of liver fibrosis based on clinicopathological analyses [9–11] and animal experiments [12].

The hepatic parenchyma is generally considered to consist of the following three distinct spaces: the intravascular space (IVS), the intracellular space, and the extravascular-extracellular space (EES) [13]. The histopathological feature of hepatic fibrosis is the expansion of the EES secondary to the deposition of collagen and matrix proteins [14]. While the measurement of the EES is challenging, the quantification of the extracellular space (ECS), which is the sum of the EES and the IVS on the CE-CT, is relatively simple. All current water-soluble conventional CT contrast materials pass freely between the EES and the IVS of the liver but are not taken up by living cells [15]. After parenteral injection, the distribution of contrast material reaches an equilibrium in which the concentration of contrast material is similar between the EES and the IVS. Images acquired at this time are commonly referred to as equilibrium-phase images. As the concentration of contrast material on CT is directly proportional to the CT attenuation, the ECV (the volume of the ECS) fraction is simply estimated as the ratio of enhancement of the liver parenchyma to enhancement of the blood pool multiplied by the difference of 1 minus the hematocrit value during the equilibrium phase.

Based on the concept of the ECV fraction, we hypothesized that the ECV fraction increases due to the expansion of the IVS in the liver of the patients with OX-induced SOS, which histopathologically presents with sinusoidal dilatation, whereas the expanding EES leads the ECV fraction to increase with the progression of liver fibrosis [12]. In the present preliminary study, we evaluated the potential of the ECV fraction to estimate the degree of SOS using histopathological analyses of specimens from patients who underwent hepatectomy after chemotherapy with OX.

## 2. Methods

### 2.1. Study Population

**Figure 1:**
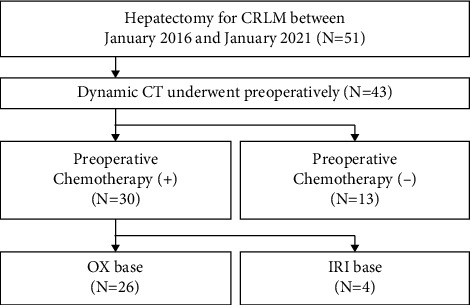
Flowchart of study enrollment. CRLM, colorectal liver metastasis; CT, computed tomography; IRI, irinotecan; and OX, oxaliplatin.

Between January 2016 and January 2021, 51 consecutive patients underwent surgical liver resection for CRLM in the Department of Surgery, Miyazaki University Hospital, Miyazaki, Japan. A retrospective review of the data for these patients was conducted. Twenty-six (51%) patients who met the inclusion criteria were included in the subsequent analysis. The inclusion criteria were as follows: systemic chemotherapy containing OX before surgery, adequate preoperative dynamic CT, within 30 days between the preoperative CT and the surgical resection, and sufficient nontumoral liver tissue to perform a representative pathological analysis (Figure 1).

This single-center, retrospective, observational study was performed at Miyazaki University Hospital and approved by the institutional ethical committee (study number: O-1132). Consent was obtained from all participants included in this study via an opt-out format.

### 2.2. CT Protocol

CT examinations were performed using a 64-row multislice CT system (IQon Spectral CT, Philips Healthcare, Eindhoven, The Netherlands) and a 320-row multislice CT system (Aquilion ONE Genesis Edition, Tokyo, Japan). After the acquisition of precontrast scans, each subject was injected with a properly selected iodinated contrast agent of one of four brands (Omnipaque, GE Healthcare, Boston, MA; Optiray, Guerbet, Paris, France; Iomeron, Eisai, Tokyo, Japan; or Iopromide, Fujifilm, Tokyo, Japan), using a Dual Shot GX7 power injector (Nemoto, Tokyo, Japan). The injection dose was 600 mg of iodine per kg of body weight, and the duration was fixed at 30 seconds; hence, the injection rate depended on the patient's body weight. Triple-phasiccontrast-enhanced scans through the abdomen were performed without a bolus tracking program. Equilibrium-phase images were obtained 180 seconds after injection. The slice thickness for the contrast-enhanced images was 2 mm. The images were saved in DICOM format and transferred to an image workstation using the SYNAPSE VINCENT software program (Fujifilm).

### 2.3. Image Analysis

**Figure 2:**
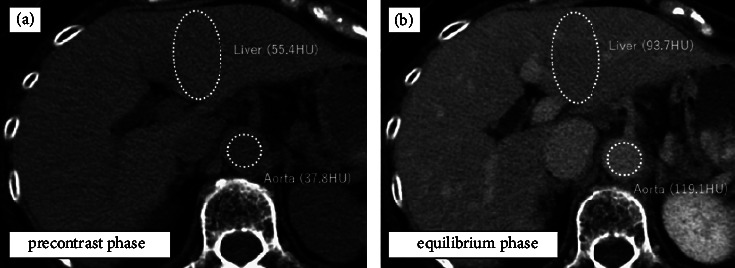
Preoperative computed tomography (CT) images from a 65-year-old woman with colorectal cancer liver metastasis after oxaliplatin-based chemotherapy. Oval regions of interest are placed and CT values are measured on the liver parenchyma and abdominal aorta in both precontrast (a) and equilibrium phase (b) images. On the basis of the hematocrit level (35.9%), ΔHU_liver_ (93.7 − 55.4 = 38.3 HU), and ΔHU_aorta_ (119.1 − 37.8 = 81.3 HU), the calculated extracellular volume fraction was (100 − 35.9) × 38.3/81.3 = 30.2%.

The image analysis was performed using electronically stored CT images without knowledge of the clinical information. CT images taken within 30 days before surgery were included in this analysis. ECV fraction in all cases was measured by a single clinician with prior guidance and agreement from a radiologist knowledgeable in ECV. Operator-defined regions of interest (ROIs) were used to measure the CT values (in Hounsfield units, HU) of the liver parenchyma and abdominal aorta on both precontrast and equilibrium-phase images. CT values of the liver parenchyma were quantified by placing an oval ROI that carefully avoided the tumor, bile duct dilatation, and intrahepatic vessels. The ROIs were placed on the 3 areas of the lateral segment, S8 and S7. The size of the ROI was measured in more than 1000 mm^2^ of liver parenchyma, with the exclusion of blood vessels. The hepatic CT-ECV fraction was calculated using the following formula [9–12]:

(1) $$\begin{array}{c} \left(100–hematocrit\left[\%\right]\right)\times \left(\frac{{∆HU}_{liver}}{{∆HU}_{aorta}}\right)\text{,} \end{array}$$

where ∆HU_liver_ and ∆HU_aorta_ represent absolute enhancement, which were the CT values on the equilibrium-phase image minus the CT values on the precontrast images of the liver parenchyma and abdominal Ao, respectively. All measurements were performed in 3 different slices and the mean values were calculated (Figure 2). Serum hematocrit levels were obtained within 48 h of the CT examination.

We calculated the splenic volume (SV) using SYNAPSE VINCENT, as described in a previous report [16]. The SV was measured before chemotherapy and after chemotherapy before resection of the CRLM. The rate of increase of the SV was calculated as follows:

(2) $$\begin{array}{c} \frac{\left(SV\;after\;chemotherapy\right)}{\left(SV\;before\;chemotherapy\right)}\times 100\text{.} \end{array}$$

### 2.4. Preoperative Assessment of the Liver Function

Preoperative serum biological data, including the platelet count (PLT, (10^9^/L)), hematocrit value (Ht, (%)), aspartate alkaline phosphatase (AST, (U/L)), alanine aminotransferase (ALT, (U/L)), and type IV collagen, were collected. An indocyanine green (ICG) test was performed as follows: a dose of 50 mg of ICG dissolved in 10 ml of sterile water was injected through a peripheral vein based on the body weight of the patient (0.5 mg/kg). The retention rate of ICG at 15 minutes (ICG R15, (%)) was calculated with the values expressed as percentages at 5, 10, and 15 minutes after injection. The AST to platelet ratio index (APRI) score [17] and a score based on the relationships among four regression coefficients (FIB-4 index) [18], which were initially used to predict fibrosis in patients suffering from hepatitis C, were calculated according to the following formulas:

(3) $$\begin{array}{c} APRI\;score=\frac{\left(AST\left[U/L\right]/{AST}_{upper\;limit\;of\;normal}\right)}{PLT\left[10^{9}/L\right]}\times 100\text{,}\\ FIB-4\;index=\frac{\left(age\left[year\right]\times AST\left[U/L\right]\right)}{\left(PLT\left[10^{9}/L\right]\times \sqrt{ALT\left[U/L\right]}\right)}\text{.} \end{array}$$

### 2.5. Histopathological Analysis

**Figure 3:**
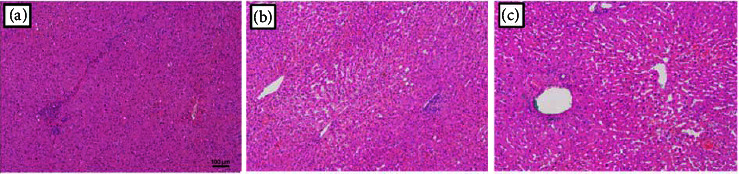
Sinusoidal congestion was graded from 0 to 3 according to the severity of the findings, as proposed in the original publication by Rubbia-Brandt et al., in which (a) grade 0 = absent, (b) grade 1 = mild (centrilobular involvement limited to one-third of the lobular surface), and (c) grade 2 = moderate (centrilobular involvement extending in two-thirds of the lobular surface). Scale bar 100 *μ*m.

**Figure 4:**
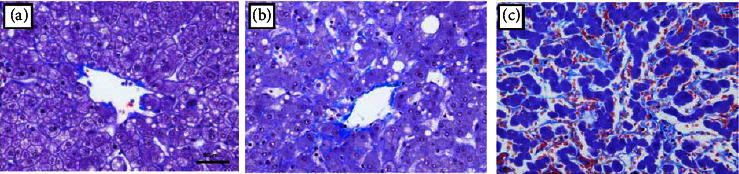
Perisinusoidal fibrosis was analyzed with Masson trichrome-stained slides and graded from 0 to 2 as follows: (a) grade 0 = absent; (b) grade 1 = mild (<50% sinusoids evaluated on 20 fields at ×200 magnification); and (c) grade 2 = moderate (>50% sinusoids evaluated on 20 fields at ×200 magnification). Scale bar 50 *μ*m.

All formalin-fixed, paraffin-embedded samples of nontumoral liver parenchyma were reviewed by one pathologist who was unaware of the clinical and biological patient data. Morphological analyses were conducted using hematoxylin and eosin (H&E)-stained slides. Sinusoidal congestion was graded from 0 to 3 according to the severity of the findings, as proposed in the original publication by Rubbia-Brandt et al. [4], in which grade 0 = absent, grade 1 = mild (centrilobular involvement limited to one-third of the lobular surface), grade 2 = moderate (centrilobular involvement extending into two-thirds of the lobular surface), and grade 3 = severe (complete lobular involvement) (Figure 3). Perisinusoidal fibrosis was also analyzed with Masson trichrome-stained slides as previously reported by Rubbia-Brandt et al. [19] and graded from 0 to 2 as follows: grade 0 = absent, grade 1 = mild (<50% sinusoids evaluated on 20 fields at ×200 magnification), and grade 2 = moderate (>50% sinusoids evaluated on 20 fields at ×200 magnification) (Figure 4).

### 2.6. Statistical Analysis

All continuous variables are expressed as the mean ± standard deviation. The Mann–Whitney *U* test was used to compare continuous variables. A one-way analysis of variance was used as a multiple comparison test. Categorical variables are summarized as numbers and percentages and were compared between groups using Fisher's exact test or the chi-squared test, as appropriate. A nonparametric receiver operating characteristic curve analysis was used to calculate the area under the receiver operating characteristic curve (AUC). Younden index is used as a criterion for selecting the optimum cutoff point. Statistical analyses were performed using StatFlex version 7 (Artech, Osaka, Japan), and *P* values of <0.05 were considered statistically significant.

## 3. Results

### 3.1. Patient Demographics

The demographic characteristics of the patients are shown in Table 1. The study population included 17 male patients and 9 female patients with a mean age of 62.1 years (range of 41–84 years). The primary lesions were colon cancer in 20 patients and rectal cancer in 6 patients. Regarding chemotherapy, 16 patients received neoadjuvant chemotherapy, while 10 patients received chemotherapy under the diagnosis of unresectable colorectal cancer and were eventually converted to surgical resection. The mean number of cycles of OX-based chemotherapy was 9.5 (range of 3–26 cycles). Thirteen patients were treated with bevacizumab, which has been reported to be effective for reducing OX-induced SOS. There were no patients with viral hepatitis.

### 3.2. Histopathological Analysis of Sinusoidal Dilatation and Perisinusoidal Fibrosis

Twenty-six specimens from the patients were graded according to the SOS classification of Rubbia-Brandt et al. as follows: grade 0, 65.4% (*n* = 17); grade 1, 15.4% (*n* = 4); and grade 2, 19.2% (*n* = 5). No specimens corresponded to grade 3. Perisinusoidal fibrosis was observed in 53.8% (*n* = 14) patients. Among them, grade 1 and grade 2 were observed in 92.9% (*n* = 13) and 7.1% (*n* = 1), respectively. Perisinusoidal fibrosis was present in 58.8% of patients without sinusoidal dilatation, 50% of patients with grade 1 sinusoidal dilatation, and 40% of those with grade 2.

### 3.3. Correlation of Imaging Parameters with Histopathological Sinusoidal Dilatation

**Figure 5:**
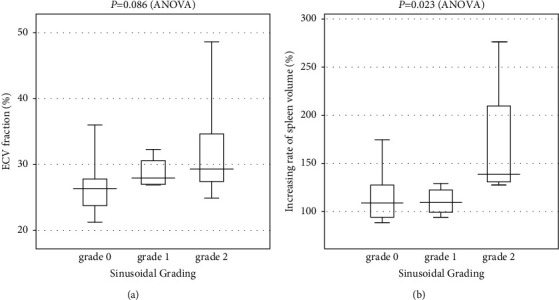
The box plots show the extracellular volume (ECV) fractions for the grading of sinusoidal obstruction syndrome (SOS) and the rate of increase of spleen volume (SV). (a) A multiple comparison test showed no significant difference in the correlation of the ECV fraction with the SOS grade (*P* = 0.086). (b) A multiple comparison test showed a significant difference in the correlation of the rate of increase of SV with the grade of SOS (*P* = 0.023).

The mean ECV fraction of each grade gradually increased from grade 0 to grade 2 sinusoidal dilatation (grade 0: 26.3 ± 3.4%, grade 1: 28.8 ± 2.5%, and grade 2: 32.1 ± 9.4%). However, a multiple comparison test showed no significant difference in the correlation of the ECV fraction with the grade of SOS (*P* = 0.086) (Figure 5(a)).

A multiple comparison test showed a significant difference in the correlation of the rate of increase of the SV with the grade of SOS (*P* = 0.023) (Figure 5(b)). The mean rate of increase of the SV in each grade was as follows: 114.2 ± 24.3% in grade 0, 110.3 ± 14.8% in grade 1, and 169.7 ± 70.5% in grade 2.

### 3.4. Predictive Factors for OX-Induced SOS

**Figure 6:**
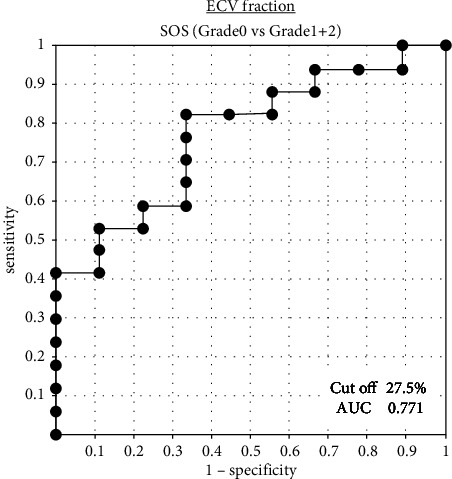
ROC curves of the extracellular volume (ECV) fraction in the prediction of sinusoidal obstruction syndrome (SOS). The cutoff value and the AUC value of the ECV fraction, which distinguishes between grade 0 SOS and grade 1 + 2 SOS, were 27.5% and 0.771, respectively.

For univariate analysis of the impact of the clinical valuables on SOS, Table 2 shows a comparison between grade 0 (*n* = 17) and grade 1 + 2 (*n* = 9). ECV fraction in grade 0 SOS was significantly lower than grade 1 + 2 SOS (26.3 ± 3.4% vs. 30.6 ± 7.0%; *P* = 0.025), while there was no significant difference in the rate of increase of the SV between grade 0 SOS and grade 1 + 2 SOS (114.2 ± 24.3% vs. 140.0 ± 24.3%; *P* = 0.159). There was no significant difference in the prevalence of perisinusoidal fibrosis between two groups (58.8% vs. 44.4%; *P* = 0.484). The cutoff value and AUC value of the ECV fraction, which distinguishes between grade 0 and grade 1 + 2, were 27.5% and 0.771, respectively (Figure 6). The cutoff value had 66.6% sensitivity and 70.6% specificity for histopathological sinusoidal dilatation.

## 4. Discussion

In SOS, histopathological changes are characterized by sinusoidal dilatation with associated hepatocyte atrophy. The microscopic change is macroscopically identified as so-called “blue liver” because the congested liver grossly shows a blue color. Rubbia-Brandt et al. [4] first reported SOS induced by OX-based chemotherapy in the nontumorous specimens of patients undergoing hepatic resection and proposed the hypothesis that an initial toxic injury to the sinusoidal endothelial cells results in sinusoidal wall disruption, which is followed by activation of hepatic stellate cells and the deposition of matrix in the sinusoids. In their retrospective study, they reported that 78% of patients who received OX had some degree of sinusoidal injury and established the invaluable histological grade classification mentioned above. A meta-analysis reporting grade 2 or greater sinusoidal injury demonstrated that those receiving OX-based regimens were at a 4.36-fold increased risk of SOS in comparison to chemotherapy-naïve control subjects [20]. According to the analysis, 17.2% of patients with OX-based chemotherapy developed moderate to severe SOS, which was similar to the frequency in our study (19.2%).

The clinical importance of SOS is reflected in the development of hepatomegaly, ascites, splenomegaly, thrombocytopenia, portal hypertension, and systemic elevation of liver enzymes [6, 21–23]. In the context of liver surgery, numerous studies have shown a negative influence of SOS on postoperative outcomes (e.g., postoperative liver failure, higher morbidity rates, and longer hospital stay) [24]. Furthermore, another study demonstrated that, over the long term, SOS could lead to early recurrence and decreased survival [25]. A histological examination of hepatic tissue is the only method that can be used to accurately determine the degree of OX-induced SOS. However, a histological examination is not feasible in the routine diagnosis of patients with suspected SOS, and it remains challenging to reliably determine the presence of SOS (which is essential to continue chemotherapy safely and better select and prepare patients before liver resection) by noninvasive imaging tests.

Several previous studies have identified predictive factors of OX-induced SOS, including the administration of six or more cycles of chemotherapy [24], the preoperative AST value [24], ICG R15 [24], APRI [26], and the FIB-4 index [26]. However, the present study found that the abovedescribed factors do not differ to a statistically significant extent between the grades of SOS. Overman et al. [6] hypothesized that changes in spleen size would reflect the extent of hepatic sinusoidal injury after OX-based chemotherapy, based on the clinical observation of prolonged thrombocytopenia and splenic enlargement in patients treated with such therapies and the understanding of the mechanism of SOS. In their retrospective study, treatment with adjuvant FOLFOX before hepatectomy resulted in an increase in spleen size in 86% of patients, and an increase in spleen size was strongly correlated with a higher grade of SOS. The present study found a significant influence of moderate (grade 2) SOS in comparison to no or mild SOS (grades 0–1) on the rate of increase of the SV. On the other hand, no influence was apparent when mild to moderate SOS (grades 1–2) was compared to no SOS (grade 0). From these results, we considered that splenic enlargement is not a suitable factor for predicting early-stage SOS because it reflects the indirect findings based on the condition in which a more advanced morphological hepatic change causes a sufficient degree of portal hypertension to cause the spleen to enlarge.

This study is the first to report that OX-induced SOS can be predicted using the ECV fraction as a noninvasive quantitative parameter calculated based on preoperative CE-CT. Although this parameter was initially described in the setting of hepatitis-related liver fibrosis [27], we herein show its usefulness in the setting of chemotherapy-related liver injury. Another important finding of our study is that CT-ECV may be able to detect the change of OX-induced SOS at an earlier time point in comparison to the rate of increase of the SV. While this novel imaging parameter has not been observed as a parameter of SOS, there is abundant evidence to support its use as a predictive factor of fibrosis in the myocardium [9], the liver [10, 27], and the pancreas [11, 28]. This study showed an important discovery in that the ECV fraction is a parameter that may be able to predict grade 0 SOS (AUC: 0.771; sensitivity: 0.667; specificity: 0.706). As a diagnostic parameter for SOS, the ECV fraction is different from the rate of increase of the SV in that the ECV fraction is a direct parameter that can be calculated based on the concept that imaging features reflect histological changes in the liver, whereas enlargement of the spleen is an indirect parameter that results from the hemodynamic change caused by the change of the hepatic parenchyma. Therefore, more useful diagnostic methods may be established when both of the parameters, which can be calculated from a single routine CE-CT scan, are well combined.

In the patients with OX-induced SOS, the fibroses were known to be proportionally associated with the severity of sinusoidal dilatation [19]. Since the ECV fraction has been correlated with tissue fibrosis in previous papers, we examined tissue fibrosis associated with the progression of SOS. Unlike the previous report, our results revealed that the prevalence of fibrosis was similar in each dilatation grade. Furthermore, no significant difference of the ECV fraction was shown between the patients with and without perisinusoidal fibrosis (28.1 ± 6.8% vs. 27.5 ± 2.8%; *P* = 0.783). The reason for concern is that the very low prevalence of severe fibrosis (grade 2 fibrosis was only 3.8% overall) had little impact on the ECV fraction. Such characteristics of the patient background might provide certain advantages in evaluating the correlation between ECV and sinusoidal dilatation.

Our study was associated with several important limitations. First, it was a retrospective study of a small number of patients. Second, we used two different CT systems. Third, when extracting CT values, some measurement errors may have occurred due to the manual ROI setting. Based on the results of this preliminary study, it will be necessary to conduct a further prospective study in a larger population using dual-energy CT. Dual-energy CT can accurately extract the iodine concentration in a specific range without subtracting the CT values of the equilibrium phase from the precontrast phase [29]. Using this imaging device, it should be possible to more accurately determine the CT-ECV fraction.

In conclusion, the ECV fraction was found to have a potential application as a noninvasive diagnostic method for determining histopathological sinusoidal injury induced by OX-based chemotherapy at an early stage. We would like to examine the clinical impact of this novel parameter on surgical outcomes after hepatectomy for CRLM in a high-quality prospective study.

## Figures and Tables

**Table 1:** Patient demographics.

Characteristic	Value
Total number of patients	26
Sex	
Male	17 (65.4%)
Female	9 (34.6%)
Age (year)	62.1 ± 11.2
Body mass index (kg/m^2^)	23.2 ± 5.2
Site of primary lesion	
Colon	20 (76.9%)
Rectum	6 (23.1%)
Chemotherapeutic regimen	
FOLFOX or CapeOX	10 (38.5%)
FOLFOX or CapeOX + BV	11 (42.3%)
FOLFOX + PV	3 (11.5%)
FOLFOXIRI + BV	2 (7.7%)
Cycle of OX-based chemotherapy	9.5 ± 6.4
Purpose of chemotherapy	
Neoadjuvant therapy	16 (61.5%)
Inoperative, converted to surgery	10 (38.5%)

BV, bevacizumab; OX, oxaliplatin; PV, panitumumab.

**Table 2:** Univariate analysis of the impact of the clinical valuables on sinusoidal obstruction syndrome.

Parameter	Grade 0	Grade 1 + 2	*P* value
	(*n* = 17)	(*n* = 9)	
Patient characteristics			
Age (years)	59.9 ± 10.8	66.1 ± 11.5	0.224
Sex (male/female)	9/8	8/1	0.067
Body mass index (kg/m^2^)	24.1 ± 6.0	21.6 ± 2.8	0.346
Chemotherapy			
Purpose (NAC/unresectable, conversion)	11/6	5/4	0.648
Cycle of OX-based chemotherapy	10.3 ± 6.8	8.1 ± 5.7	0.471
Use of bevacizumab (yes/no)	10/7	3/6	0.216
Preoperative biologic data			
AST (U/L)	23.4 ± 6.1	32.2 ± 13.3	0.100
Hematocrit (%)	38.7 ± 5.1	38.1 ± 5.7	0.628
Type IV collagen (ng/mL)	6.32 ± 1.78	8.26 ± 4.28	0.477
ICG R15 (%)	12.0 ± 11.3	12.3 ± 4.6	0.162
APRI	0.33 ± 0.12	0.53 ± 0.31	0.100
FIB-4 index	1.67 ± 0.84	2.28 ± 0.85	0.090
Histopathological finding			
Fibrotic grading (Grade 0/1 or 2)	7/10	5/4	0.484
Imaging parameter			
ECV fraction (%)	26.3 ± 3.4	30.6 ± 7.0	0.025
Increasing rate of SV (%)	114.2 ± 24.3	140.0 ± 56.8	0.159

AST, aspartate alkaline phosphatase; APRI, AST to platelet ratio index; ECV, extracellular volume; ICG R15, retention rate of indocyanine green at 15 min; SV, spleen volume.

## Data Availability

The datasets generated and/or analyzed during the current study are available from the corresponding author on reasonable request.

## Abbreviations

CE-CT: Contrast-enhanced computed tomography
CRLM: Colorectal liver metastasis
ECS: Extracellular space
ECV: Extracellular volume
EES: Extravascular-extracellular space
HU: Hounsfield unit
IVS: Intravascular space
OX: Oxaliplatin
ROIs: Regions of interest
SOS: Sinusoidal obstruction syndrome
SV: Splenic volume.
